# Pregnancy and obstetric outcomes of dichorionic triamniotic triplet pregnancies with selective foetal reduction after assisted reproductive technology

**DOI:** 10.1186/s12958-024-01199-6

**Published:** 2024-03-15

**Authors:** Shuhua Liu, Qianhua Xu, Dehong Liu, Qiuru Li, Jingyu Qian, Bin Zhang, Xianxia Chen

**Affiliations:** 1 Department of Obstetrics and Gynecology, Hefei Maternity Child Health Hospital, Hefei, 230000 China; 2 Department of Obstetrics and Gynecology, Anhui Women and Children’s Medical Center, Hefei, 230000 China; 3 Reproductive Medicine Center, Department of Obstetrics and Gynecology, The First Affiliated Hospital of Anhui Medical University, Hefei, 230032 China

**Keywords:** Dichorionic diamniotic (DCDA), Dichorionic triamniotic (DCTA), Triplet pregnancy, Monochorionic diamniotic (MCDA), Reduction, Assisted reproductive technology

## Abstract

**Background:**

It is generally beneficial and recommended that dichorionic triamniotic (DCTA) triplet pregnancies be reduced to monochorionic (MC) twin or singleton pregnancies after assisted reproductive technology (ART). However, some infertile couples still have a firm desire to retain twins. For this reason, the best foetal reduction strategies need to be available for infertile couples and clinicians. Given that data on the elective reduction of DCTA triplet pregnancies to twin pregnancies are scarce, we investigated the outcomes of elective reduction of DCTA triplet pregnancies through the retrospective analysis of previous data.

**Method:**

Patients with DCTA triplet pregnancies who underwent elective foetal reduction between January 2012 and June 2020 were recruited. A total of 67 eligible patients with DCTA triplet pregnancies were divided into two groups: a DCTA-to-dichorionic diamniotic (DCDA) twin group (*n* = 38) and a DCTA-to-monochorionic diamniotic (MCDA) twin group (*n* = 29); the basic clinical data of the two groups were collected for comparison.

**Results:**

Compared with the DCDA-to-MCDA twin group, the DCTA-to-DCDA twin group had lower rates of complete miscarriage (7.89% versus 31.03%, *p* = 0.014), early complete miscarriage (5.26% versus 24.14%, *p* = 0.034), late preterm birth (25.71% versus 65.00%, *p* = 0.009) and very low birth weight (0 versus 11.11%, *p* = 0.025). In addition, the DCTA-to-DCDA twin group had higher rates of full-term delivery (65.71% versus 25.00%, *p* = 0.005), survival (92.11% versus 68.97%, *p* = 0.023), and taking the babies home (92.11% versus 68.97%, *p* = 0.023) than did the DCTA-to-MCDA twin group. In terms of neonatal outcomes, a significantly greater gestational age (38.06 ± 2.39 versus 36.28 ± 2.30, *p* = 0.009), average birth weight (3020.77 ± 497.33 versus 2401.39 ± 570.48, *p <* 0.001), weight of twins (2746.47 ± 339.64 versus 2251.56 ± 391.26, *p* < 0.001), weight of the larger neonate (2832.94 ± 320.58 versus 2376.25 ± 349.95, *p* < 0.001) and weight of the smaller neonate (2660.00 ± 345.34 versus 2126.88 ± 400.93, *p* < 0.001) was observed in the DCTA-to-DCDA twin group compared to the DCTA-to-MCDA twin group.

**Conclusion:**

The DCTA-to-DCDA twin group had better pregnancy and neonatal outcomes than the DCTA-to-MCDA twin group. This reduction approach may be beneficial for patients with dichorionic triamniotic triplet pregnancies who have a strong desire to have DCDA twins.

## Introduction

In recent years, the incidence of multiple pregnancies has increased significantly due to ART [1–3]. Maternal complications, including hyperemesis gravidarum, gestational diabetes, gestational hypertension, anaemia, postpartum haemorrhage, caesarean section, and postpartum depression, are more common in women with multiple pregnancies than in those with singleton pregnancies [4–7]. Moreover, neonatal complications have increased, and the most concerning complications are preterm birth, preterm morbidity, and perinatal mortality [8, 9]. Multiple pregnancies are reportedly associated with an increase in cerebral palsy in children; triplets are 47 times more likely to have cerebral palsy than singletons are, and twins are 8 times more likely to have cerebral palsy than singletons are [10].

DCTA triplet pregnancy is a special type of triplet pregnancy, and our previous study revealed that the incidence of DCTA was 1.24% among all clinical pregnancies conceived through ART [11]. In addition to the common complications of multiple pregnancies, DCTA triplet pregnancy is associated with specific complications caused by placental vascular anastomosis [12, 13], such as twin anaemia polycytaemia sequence (TAPS), twin-twin transfusion syndrome (TTTS), selective intrauterine growth restriction (SIGR), and twin reversed arterial perfusion sequence (TRAPs), which can affect infant and maternal morbidity and mortality.

Studies have shown that multifoetal pregnancy reduction (MFPR) improves the outcomes of multiple pregnancies, especially high-order multiple pregnancies [14–17]. There are three strategies for DCTA triplet pregnancy reduction: reduction of the MCDA twins and retaining the singleton; reduction of the singleton and retaining the MCDA twins, or reduction of one of the MCDA twins and continuing the pregnancy as a DCDA twin pregnancy [18]. However, most studies suggest that the reduction of MCDA twin pregnancies to retain singleton pregnancies is a safe and feasible strategy for treating DCTA triplet pregnancies [19–24]. However, there are still many infertile couples who want to retain twins, so better strategies are needed to avoid obstetric risks and satisfy their strong desires.

Currently, studies on the clinical outcomes of foetal selective reduction in DCTA triplet pregnancies are scarce. Moreover, in these sporadic studies, no exact reduction strategy has been given. A comparative study of only 9 DCTA-to-DCDA twin pregnancies and 18 DCTA-to-TCTA twin pregnancies showed no difference in preterm birth rates, term birth rates, gestational age, and taking the babies home rate between the two groups, except in the late miscarriage rate, which was higher in the DCTA-to-DCDA twin group; the study concluded that the DCTA-to-DCDA twin pregnancy strategy was feasible [22]. However, studies of DCTA-to-MCDA twin pregnancies showed that, compared with expectant management, DCTA-to-MCDA twin pregnancies had lower miscarriage rates, greater live birth rates and greater taking the babies home rates; moreover, these studies did not compare with TCTA-to-DCDA twin pregnancies or DCTA-to-DCDA twin pregnancies [23]. These authors suggested that the relatively high likelihood of miscarriage and perinatal mortality for DCTA-to-MCDA twin pregnancies is related to the abnormal and dangerous placental structure leading to serious complications, such as TTTS, SIGR, TRAP, TAPS, umbilical cord compression, and entanglement, rather than to the reduction itself. They suggested that reduction to a singleton pregnancy with a separate placenta may be an acceptable reduction strategy compared to reduction to one MCDA twin; although this strategy carries a special risk of complications associated with MCDA twin pregnancies, it has a relatively low miscarriage rate [23]. However, a recent study of DCTA-to-DCDA twin pregnancies showed that compared to DCTA-to-MCDA twin pregnancies, DCTA-to-DCDA twin pregnancies had lower rates of early miscarriage, preterm birth, and caesarean section, while the newborn birth weight, full-term birth rate, and taking the babies home rate were greater [25].

To satisfy the desire of patients with infertility to retain twins, this study aimed to investigate the DCTA pregnancy outcomes after elective foetal reduction to identify options to avoid MCDA pregnancy complications and meet the strong desires of these couples and to provide a safe and feasible reduction strategy for clinical practice.

## Materials and methods

### Subjects

We retrospectively analysed study participants with DCTA triplet pregnancies between 2012 and 2020 who underwent elective embryo reduction at 6–8 weeks after embryo transfer at our medical centre. A total of 67 patients with DCTA triplet pregnancies were enrolled and divided into two subgroups: a DCTA-to-DCDA twin group (*n* = 38) and a DCTA-to-MCDA twin group (*n* = 29). This study was approved by the Ethics Review Committee of the First Affiliated Hospital of Anhui Medical University (PJ20180707) and was carried out in accordance with the principles of the Declaration of Helsinki. Written informed consent was obtained from all patients after an explanation of the study was provided.

#### MFPR procedures

DCTA triplet pregnancies were diagnosed by transvaginal ultrasound at 6–8 weeks after embryo transfer. The attending physician introduced the infertile couple to multiple pregnancies and the potential risks of MCDA twin pregnancies to the foetus and the mother; the surgical procedures and risks were also described, and the final reduction in the number of foetuses and the selective foetal reduction strategy were decided upon by the patient after consultation. Patients signed the informed consent form regarding the risks of foetal reduction surgery.

MFPR was performed 6–8 weeks after embryo transfer. Foetal reduction surgery was performed by an experienced doctor under transvaginal ultrasound guidance. The surgery involved puncturing and aspirating the selected embryo site without administering any medication. Pregnant women were given antibiotics to prevent infection 1 week before surgery, and antibiotics to prevent infection and luteal support were given according to the patient’s condition after surgery. The remaining foetuses were examined on the 1st and 5th postoperative days.

### Statistical analysis

The data were analysed with SPSS software (SPSS, Inc., Chicago, IL, version 24.0). Variables that fit a normal distribution are expressed as the mean ± standard deviation (SD). The t test, the chi-square test and Fisher’s exact test were used for differences between normally distributed data and differences in percentages between two groups. A p value < 0.05 was considered to indicate a statistically significant difference.

## Results

### Patient demographic characteristics

A total of 67 eligible patients with DCTA triplet pregnancies were divided into two groups: a DCTA-to-DCDA twin group (*n* = 38) and a DCTA-to-MCDA twin group (*n* = 29). The patients conceived through embryo transfer (ET) after undergoing ART. The demographic characteristics of the participants are presented in Table 1. Maternal age, the interval between transplantation and MFPR, BMI, the duration of infertility, the infertility type, F-ET status, insemination methods and the number of embryos transferred were not significantly different between the two groups (all *p* > 0.05).

**Table 1:** demographic characteristics

	DCTA to MC twins (*n* = 29)	DCTA to DC twins (*n* = 38)	P value
Maternal age (years)	30.72 ± 4.46	29.47 ± 2.59	0.185
Interval between transplantation and MFPR (days)	38.31 ± 4.52	38.71 ± 4.32	0.714
BMI (kg/m^2^)	20.85 ± 2.61	21.80 ± 2.88	0.167
Duration of infertility (years)	4.14 ± 2.37	3.68 ± 2.38	0.442
Infertility type			
Primary, n (%)	23/29	29/38	1.000
Secondary, n (%)	6/23	9/38	
FET (N)			
not-used (%)	11/29	13/38	0.801
used (%)	18/29	25/38	
Insemination methods			
ICSI, n (%)	14/29	14/38	0.454
IVF, n (%)	15/29	24/38	
Number of embryos transferred	2 (2.00,2.00)	2 (2.00,2.00)	1.000

No significant difference was found between the three sets of data in the DCTA group

### Differences in pregnancy and delivery outcomes between the DCTA-to-DCDA twin group and DCTA-to-MCDA twin group

The pregnancy and delivery outcomes are presented in Table 2. Three complete miscarriages occurred before 28 weeks in the DCTA-to-DCDA twin group, and nine complete miscarriages occurred in the DCTA-to-MCDA twin group. In the end, a total of 18 monochorionic (MC) singletons and 17 other fraternal twins were born to women in the DCTA-to-DCDA twin group, and 4 MC singletons and 16 MCDA twins were born to women in the DCTA-to-MCDA twin group. The DCTA-to-DCDA twin group had lower rates of complete miscarriage (7.89% versus 31.03%, *p* = 0.014), early complete miscarriage (5.26% versus 24.14%, *p* = 0.034), late preterm birth (25.71% versus 65.00%, *p* = 0.009) and very low birth weight (0 versus 11.11%, *p* = 0.025) than did the DCTA-to-MCDA twin group. In addition, the DCTA-to-DCDA twin group had higher rates of full-term delivery (65.71% versus 25.00%, *p* = 0.005), survival (92.11% versus 68.97%, *p* = 0.023), and taking the babies home (92.11% versus 68.97%, *p* = 0.023) than did the DCTA-to-MCDA twin group. There was no difference in the incidence of obstetric complications between the two groups (all *p* > 0.05) (Table 2).

**Table 2:** Comparing the pregnancy and delivery outcomes

	DCTA to MCDA twins (*n* = 29)	DCTA to DCDA twins (*n* = 38)	P value
Pregnancy outcomes			
Complete miscarriage rate (%)	9/29 (31.03)	3/38 (7.89)	**0.014**
Early complete miscarriage rate (< 12 weeks)	7/29 (24.14)	2/38 (5.26)	**0.034**
Late complete miscarriage rate (12–28 weeks)	2/29	1/38	0.574
Premature delivery rate (%)	15/20	12/35	0.005
Early premature delivery (28–34 weeks)	2/20	3/35	1.000
Late premature delivery (34-37weeks)	13/20 (65.00)	9/35 (25.71)	**0.009**
Full-term delivery (%)	5/20 (25.00)	23/35 (65.71)	**0.005**
Survival rate (%)	20/29 (68.97)	35/38 (92.11)	**0.023**
Take baby home rate (%)	20/29 (68.97)	35/38 (92.11)	**0.023**
One survival (%)	4/20 (20.00)	18/35 (51.43)	**0.044**
Twin survival (%)	16/20 (80.00)	17/35 (48.57)	
Cesarean section rate (%)	17/20	25/35	0.333
Obstetric complications			
Total obstetric complications	9/20	10/35	0.250
Gestational hypertension	2/20	3/35	1.000
Gestational diabetes	2/20	4/35	1.000
Hyperthyroidism	1/20	0/35	0.364
Hypothyroidism	1/20	1/35	1.000
Postpartum hemorrhage	1/20	2/35	1.000
Thrombocytopenia	0	0	/
Placenta previa	1/20	0/35	0.364
Placenta residue	1/20	0/35	0.364

DCTA: Dichorionic triamniotic riplet pregnancy;Dichorionic twins:DCDA;

Bold values was statistically signifificant (*P* < 0.05)

**Table 3:** Comparing the neonatal outcomes

	DCTA to MCDA twins (*n* = 29)	DCTA to DCDA twins (*n* = 38)	P value
Gestational week (weeks)	36.28 ± 2.30	38.06 ± 2.39	**0.009**
Average birth weight (g)	2401.39 ± 570.48	3020.77 ± 497.33	**< 0.001**
Percentage of boys (%)	17/36	25/52	1.000
Very low birth weight (less than 1,500 g)	4/36 (11.11)	0/52 (0.00)	**0.025**
Weight of twins (g)	2251.56 ± 391.26	2746.47 ± 339.64	**< 0.001**
Weight of larger neonate in twins (g)	2376.25 ± 349.95	2832.94 ± 320.58	**< 0.001**
Weight of smaller neonate in twins (g)	2126.88 ± 400.93	2660.00 ± 345.34	**< 0.001**
Weight difference of twins (g)	249.38 ± 205.05	172.94 ± 83.65	0.181
Neonatal morbidities			
Total neonatal morbidities	10/36	10/47	0.492
Ventricular septal defect	2/36	1/47	0.576
Renal dysplasia	1/36	0/47	0.434
Neonatal pneumonia	2/36	3/47	NS
Neonatal jaundice	3/36	2/47	0.648
Neonatal hypoglycemia	2/36	3/47	NS
Neonatal infections	0/36	1/47	NS

DCTA: Dichorionic triamniotic riplet pregnancy;Dichorionic twins:DCDA; MCDA:monochorionic diamniotic twins

Bold values was statistically signifificant (*P* < 0.05)

### Differences in neonatal outcomes between the DCTA-to-DCDA twin group and DCTA-to-MCDA twin group

In Table 3, a total of 52 newborns were born to women in the DCTA-to-DCDA twin group, including 17 fraternal twins and 18 MC singletons, while a total of 36 newborns were born to women in the DCTA-to-MCDA twin group, including 16 MCDA twins and 4 MC singletons. As expected, a significantly greater gestational age (38.06 ± 2.39 versus 36.28 ± 2.30, *p* = 0.009), average birth weight (3020.77 ± 497.33 versus 2401.39 ± 570.48, *p <* 0.001), weight of twins (2746.47 ± 339.64 versus 2251.56 ± 391.26, *p* < 0.001), weight of the larger neonate (2832.94 ± 320.58 versus 2376.25 ± 349.95, *p* < 0.001) and weight of the smaller neonate (2660.00 ± 345.34 versus 2126.88 ± 400.93, *p* < 0.001) were observed in the DCTA-to-DCDA twin group compared to the DCTA-to-MCDA twin group. No significant difference was observed in the incidence of neonatal morbidities between the two groups (all *p* > 0.05).

### Spontaneous foetal reduction after DCTA reduction to twins

Table 2 shows that before 28 weeks, there were 3 complete miscarriages in the DCTA-to-DCDA twin group and 9 in the DCTA-to-MCDA twin group after DCTA reduction to twins. There were a total of 24 spontaneous foetal reductions in the DCTA-to-DCDA twin group, of which 18 involved the reduction of another foetus after MCDA reduction, while 22 pregnancies in the DCTA-to-MCDA twin group were spontaneously reduced; 4 of these cases were among the MCDA twins (Table 4). The timing of spontaneous foetal reduction occurred mostly within 12 weeks in the DCTA-to-DCDA twin and DCTA-to-MCDA twin groups (81.82% versus 91.67%, *p* = 0.405).

**Table 4:** Spontaneous fetal reduction after DCTA reduction to twin

Spontaneous fetal reduction	DCTA to MCDA twins (*n* = 22)	DCTA to DCDA twins (*n* = 24)	P value
From MC twin	22	24	
Early spontaneous fetal reduction (< 12 weeks)	18/22 (81.82)	22/24 (91.67)	0.405
Late spontaneous fetal reduction (12–28 weeks)	4/22 (18.18)	2/24 (8.33)	0.405
During labor	0	0	/
From MC singleton	0	0	/

DCTA: Dichorionic triamniotic riplet pregnancy; Dichorionic twins:DCDA

## Discussion

It is well established that DCTA triplet pregnancies can be detrimental to mothers and babies. Therefore, to minimize the occurrence of multiple pregnancies, we recommend that the number of embryos transferred not exceed two day 3 cleavage embryos or one blastocyst in the first cycle. Nevertheless, multiple pregnancies are unavoidable. Our previous investigation revealed that a maternal age < 35 years, blastocyst transfer and the use of intracytoplasmic sperm injection were risk factors for DCTA triplet pregnancy [11]. In addition, it has also been reported that the laboratory environment, medium conditions [26], genetic factors [27], and zona pellucida operation [28, 29] may also be factors affecting the development of DCTA triplets.

To reduce the risk of multiple pregnancies, MFPR is often used as an option to improve pregnancy outcomes. According to the guidance of the Chinese Reproductive Commission, multiple pregnancies are recommended to be reduced to singleton pregnancies as much as possible to reduce adverse pregnancy outcomes [30]. However, there are still some infertile patients who strongly desire to retain twins. In view of the findings of this study and previous literature reports, the reduction of a DCTA triplet pregnancy to a DCDA twin pregnancy is a feasible foetal reduction strategy without considering twin survival.

For the MFPR strategy, two aspects should be considered when retaining a singleton pregnancy or a twin pregnancy (DCDA or MCDA). First, our previous studies suggest that the rate of miscarriage and premature delivery in DCTA triplet pregnancies reduced to twin pregnancies is greater than that in DCTA triplet pregnancies reduced to MC singleton pregnancies, while the live birth rate and the rate of taking the babies home are significantly lower than those in MC singleton pregnancies [31]. Additionally, the perinatal mortality [32] and neonatal morbidity [33, 34] rates are also greater than those in the MC singleton group. Additionally, twin pregnancies are associated with 3 to 7 times greater perinatal mortality and morbidity than singleton pregnancies [35]. The high mortality and morbidity are due to the higher incidence of prenatal complications, preterm birth, and uteroplacental insufficiency [35]. Therefore, DCTA triplet pregnancies should be reduced to singleton pregnancies whenever possible. Second, compared with DCDA twin pregnancies, MCDA twin pregnancies have significantly greater rates of stillbirth and neonatal mortality: 44.4 versus 12.2 per 1000 live births [relative risk (RR): 3.6; 95% CI 2.6–5.1] and 32.4 versus 21.4 per 1000 live births (RR: 1.5; 95% CI: 1.04–2.2), respectively [36]. Similarly, studies have shown that the perinatal mortality rate of MCDA twin pregnancies is more than twice that of DCDA twin pregnancies (11.6%, versus 5.0%) [37]. After 32 weeks, the risk of intrauterine death was significantly greater for MCDA twin pregnancies than for DCDA twin pregnancies (hazard ratio 8.8, 95% CI 2.7–28.9), and in most cases of intrauterine death, there were no prenatal signs of impaired foetal conditions [37]. In addition, the incidence of congenital malformations in MCDA twin pregnancies is 2.5 times greater than that in DCDA twin or singleton pregnancies [38]. This study revealed a greater complete miscarriage rate (34.48% versus 7.89%, *p* = 0.011) and late preterm birth rate (65.00% versus 25.71%, *p* = 0.009); a shorter gestational age at delivery (36.28 ± 2.30 versus 38.06 ± 2.39, *p* = 0.009); and a lower average birth weight (2401.39 ± 570.48 versus 3020.77 ± 497.33, *p* < 0.001). Previous studies have suggested that DCDA twin pregnancies are associated with longer gestational weeks and better neonatal outcomes. The median gestational age of DC twins was 1 week longer than that of MCDA twins, with a mean birth weight that was 221 g higher. Birth weight disagreement (> 20%) was more common in MCDA twin pregnancies than in DCDA twin pregnancies (odds ratio [odds] 1.23, 95% CI 0.97–1.55). MCDA twin pregnancies had a greater incidence of necrotizing enterocolitis (NEC), adjusted for age and weight at birth (odds ratio 4.05, 95% CI 1.97–8.35), and there was an upwards trend in neurological morbidity in MCDA twin pregnancies [37]. This study showed that the gestational age, average birth weight, and taking the babies home rate were significantly greater in the DCTA-to-DCDA twin group than in the DCTA-to-MCDA twin group, and the complete miscarriage rate and late preterm birth rate were lower in the DCTA-to-DCDA twin group than in the DCTA-to-MCDA group. In addition, although our study did not suggest any abnormalities in the neonatal nervous system, this does not mean that the neurological damage of the remaining surviving foetuses after foetal reduction was low, which may be related to the small amount of data in our study. Severe neurological injury has been reported in 18–24% of foetuses that survive intrauterine foetal death [39, 40]. Therefore, for infertile couples who strongly desire to preserve twins, based on the previous literature and this study, the reduction of DCTA triplet pregnancies to DCDA twin pregnancies is a feasible option.

In the first trimester, after DCTA triplet pregnancies were reduced to DCDA and MCDA twin pregnancies, there was a spontaneous foetal reduction in both groups but no difference between the groups, which may be related to the following mechanisms. First, damage and infection due to foetal reduction occur within two weeks of embryo reduction. Second, the necrotic embryonic tissue that causes the inflammatory response is reabsorbed, which may cause miscarriage of the remaining foetus at a later stage [41–43]. In this regard, the study noted that for the reduction of a DCTA triplet pregnancy to an MC singleton pregnancy or expectant management, spontaneous foetal reduction can occur [25, 31, 32]. Therefore, for spontaneous foetal reduction, we should fully inform and communicate with infertile couples about the pros and cons of MFPR. Several studies suggest that the selective reduction of one MCDA twin is infeasible due to vascular anastomoses of 96% of vessels in the single placental bed [44]. However, this study showed that the reduction of a DCTA triplet pregnancy to an MCDA twin pregnancy is associated with a greater complete miscarriage rate than the reduction of a DCTA triplet pregnancy to a DCDA twin pregnancy, and there was no difference in spontaneous foetal loss rates. Other studies reported similar results (21.4% versus 0%, *P* = 0.004). Therefore, we believe that the reduction of a DCTA triplet pregnancy to a DCDA twin pregnancy is a viable strategy for infertile couples who desire to preserve twins and avoid or minimize obstetric risks.

In addition, we chose to evaluate the timing of foetal reduction at 6 to 8 weeks after embryo transfer, i.e., between gestational weeks 8 and 11, because this gestational age is accurate for identifying chorions [45]. These findings are consistent with those of Bora et al., who reported that transvaginal ultrasound had very high agreement in diagnosing chorionic and amniotic pregnancies in twin-pregnant women at 7 to 9 weeks and 11 to 14 weeks, indicating a similar level of accuracy [46]. Potassium chloride injection may cause damage to the remaining foetus through vascular anastomosis in the placenta [47]. The method we used for reduction was intracardiac puncture and aspiration. This approach has also been shown in several studies to be a feasible and effective MFPR method for MCDA twin reduction [22, 48–50]. In addition, there have been reports of foetal reduction modalities, such as the removal of a monochorionic twin by the foetal space laser technique, but this may also endanger the remaining twin [51]. Chaveeva et al. reported 61 pregnant women whose DCTA pregnancies were reduced to dichorionic twin pregnancies by intrafoetal laser ablation. While 3% of miscarriages occurred after foetal reduction, nearly half of all cases occurred within two weeks of reduction [52].

This was a single-centre retrospective analysis and may be partially statistically insignificant due to data volume limitations. In addition, eligible patients were not randomly assigned to each group, so there may be bias in the results of the study. Because of the wishes of infertile couples and ethical considerations, some couples may choose whether to undergo elective foetal reduction, so this study is unlikely to be suitable for a randomized controlled trial. In addition, some of the data were collected through telephone interviews, so the data may be susceptible to recall bias. However, there are several advantages to our research. Because there are very few studies on post-femto-twin DCTA removal, this study may supplement the referential literature on the results of DCTA foetal reduction to twin pregnancies (DCDA and MCDA) for reproductive clinicians to the greatest extent possible, and the inclusion criteria and statistical methods of this study were rigorous.

## Conclusions

This study showed that the reduction of DCTA triplet pregnancies to DCDA twin pregnancies at 6–8 weeks after embryo transfer was associated with a relatively better pregnancy outcome than was reduction to MCDA twin pregnancies, and this outcome was acceptable. This reduction strategy may be an appropriate option for patients with DCTA triplet pregnancies who have a strong desire to preserve fraternal twins.

## Data Availability

The datasets used and/or analyzed during the current study are also available from the corresponding author on reasonable request.

## Abbreviations

MFPR: multifetal pregnancy reduction
DCTA: dichorionic triamniotic triplet pregnancy
MC: monochorionic twin
ART: assisted reproductive technology
DCDA: dichorionic diamniotic
MCDA: monochorionic diamniotic
TAPS: anaemia polycytaemia sequence
TTTS: twin-to-twin transfusion syndrome
SIGR: selective intrauterine growth restriction
TAPS: twin anemia-polycythemia sequence
TRAPs: twin reversed arterial perfusion sequence
ET: embryotransplant
FET: frozen embryo transplant
BMI: body mass index
LBW: low birth weight
VLBW: very low birth weight
